# Real-World Implementation of Next-Generation Sequencing in Sarcoma: Molecular Insights and Therapeutic Outcomes †

**DOI:** 10.3390/medsci14010046

**Published:** 2026-01-17

**Authors:** Tasnim Diab, Ali Tarhini, Ghina Jaber, Chris Raffoul, Nijad Zeineddine, Lara Kreidieh, Ali Hemade, Mounir Barake, Said Saghieh, Rami Mahfouz, Hazem I. Assi

**Affiliations:** 1 Department of Internal Medicine, Division of Hematology and Oncology, Naef K Bassile Cancer Institute, American University of Beirut Medical Center, Beirut P.O. Box 11-0236, Lebanon; td19@aub.edu.lb (T.D.); gj17@aub.edu.lb (G.J.); alihemade3@gmail.com (A.H.); mounir.97@hotmail.com (M.B.); 2 Faculty of Medicine, American University of Beirut, Beirut P.O. Box 11-0236, Lebanon; aht13@mail.aub.edu (A.T.); cgr04@mail.aub.edu (C.R.); nsz12@mail.aub.edu (N.Z.); lyk09@mail.aub.edu (L.K.); 3 Department of General Surgery, American University of Beirut Medical Center, Beirut P.O. Box 11-0236, Lebanon; ss15@aub.edu.lb; 4 Department of Pathology and Laboratory Medicine, American University of Beirut Medical Center, Beirut P.O. Box 11-0236, Lebanon; rm11@aub.edu.lb

**Keywords:** sarcoma, next-generation sequencing, precision oncology, molecular profiling, targeted therapy, real-world evidence, MENA Region, genomic alterations

## Abstract

**Background:** Sarcomas are rare, aggressive malignancies with limited therapeutic options in advanced stages. This is the first real-world study in the MENA region evaluating the clinical utility of Next-Generation Sequencing (NGS) in guiding sarcoma treatment and improving outcomes. **Methods:** We retrospectively reviewed sarcoma patients who underwent NGS at a major referral center (2021–2024), comparing clinical and molecular outcomes between those who received NGS-based treatment adjustments (NBTA) and those who did not. **Results:** Seventy-eight patients were included (60% male; median age 44 years). Soft tissue sarcomas accounted for 70.5% of cases (n = 55), while bone sarcomas represented 29.5% (n = 23). Prior to NGS, 64.1% of patients had received a median of one line of systemic therapy. NGS was performed late in the disease course in 73% of cases. At least one mutation was detected in 87% (median 3 mutations). Targetable alterations were identified in 33% at the time of testing, rising to 42% with updated genomic knowledge and therapeutic advances. Overall, 20.5% received NBTA. Among non-NBTA patients, 67% had no actionable targets, 17% had no detectable mutations, and 16% were ineligible due to cost, limited access, or clinical deterioration. Tumor Mutational Burden was low in 79%, intermediate in 19%, and high in 2%, and all tumors were microsatellite stable. Patients receiving NBTA had a longer median Progression-Free Survival (9 vs. 2 months; *p* = 0.023). Median Overall Survival was longer in the NBTA group (74 vs. 48 months), though not statistically significant (*p* = 0.207). Genomic alterations were subtype-specific: EWSR1 rearrangements (Ewing and Desmoplastic small round cell tumors), CDK4 and MDM2 amplifications (Liposarcoma and Osteosarcoma), TP53 and RB1 mutations (Leiomyosarcoma), CDKN2A/B deletions (Undifferentiated Pleomorphic Sarcoma and Chondrosarcoma), and SS18 rearrangements (Synovial Sarcoma). **Conclusions:** Genomics-guided therapy in sarcoma is feasible and impactful. Expanding timely access to molecular profiling is essential for advancing precision oncology in the MENA region.

## 1. Introduction

Next-generation sequencing (NGS) has transformed cancer research by enabling comprehensive molecular profiling of tumors, allowing for the detection of genetic mutations and potential therapeutic targets. In sarcomas, a rare and heterogeneous group of malignant tumors originating from mesenchymal tissues such as bone, muscle, and fat, NGS holds particular promise. Sarcomas account for approximately 1% of adult cancers and are classified into soft tissue sarcomas (STS) and bone sarcomas (BS) [1,2]. These tumors often present a poor prognosis, with about one-third of patients experiencing disease-related mortality [3]. Their heterogeneity, with over 100 subtypes, complicates the prediction of outcomes such as metastasis, recurrence, and overall survival [2,4].

Traditional diagnostic and management approaches rely heavily on tissue biopsy, which provides only a static view of tumor biology and often fails to capture spatial and temporal heterogeneity. Moreover, actionable biomarkers to guide treatment selection or monitor disease dynamics are lacking for most sarcoma subtypes [5]. To address these challenges, comprehensive molecular characterization has emerged as a critical tool. The Cancer Genome Atlas (TCGA) profiled 206 adult soft-tissue sarcomas, revealing low rates of highly recurrent somatic mutations but pervasive copy-number alterations and diverse structural variants, highlighting complex tumor evolution and the need for tailored strategies [6].

Enterprise-wide implementation of NGS in oncology has demonstrated that universal clinical tumor sequencing is feasible and can accelerate therapeutic advances when integrated with robust informatics and multidisciplinary review [7]. In a real-world cohort spanning 50,000 samples, comprehensive genomic profiling (CGP) achieved a 96.2% success rate, with clinically actionable alterations identified in nearly half of cases across tumor types [8]. Specifically in sarcoma, CGP in over 100 diverse cases uncovered druggable alterations ranging from PI3K pathway mutations to kinase fusions, identified in 41% of tumors, with about 7.5% of patients experiencing clinical benefit when treated with matched therapies [9].

For example, in leiomyosarcoma, a “*BRCA*-like” mutational pattern suggests sensitivity to PARP inhibitors (e.g., Olaparib) combined with cisplatin. Additionally, targeted therapies for oncogenic fusions, such as Larotrectinib for *NTRK*-rearranged sarcomas, are under investigation, as are epigenetic vulnerabilities, such as *BET* inhibitors for rhabdomyosarcoma and synovial sarcoma [3]. While some progress has been made, such as the use of tyrosine kinase inhibitors (TKIs) like imatinib for gastrointestinal stromal tumors (GISTs), the scarcity of effective targeted therapies for most sarcoma subtypes highlights the need for continued exploration of molecular and genomic drivers. Immunotherapy, including immune checkpoint inhibitors such as anti-PD-1 and anti-PD-L1, has shown limited success, though tumor mutational burden (TMB) and microsatellite instability (MSI) are emerging as potentially useful biomarkers [3,10].

Initially, access to NGS was limited to research settings due to high cost and technical complexity, but declining costs and technological advances have now made it increasingly accessible in routine practice. This shift has expanded opportunities to identify actionable mutations, optimize patient care, and support clinical trial enrollment. Despite these advances, the clinical application of NGS in sarcoma remains underexplored, particularly in the Middle East and North Africa (MENA) region, where access to molecular profiling and targeted therapies is restricted. This study aims to evaluate the role of NGS in sarcoma management at the American University of Beirut Medical Center (AUBMC), a leading tertiary referral center in Lebanon.

This study will identify sarcoma patients who underwent NGS, determine the frequency and types of actionable mutations found, assess treatment modifications and their clinical impact, evaluate patient access to targeted therapies, and analyze outcomes such as response rates, morbidity, and survival.

A unique feature of this study is its focus on how molecular profiling translates into therapy matching and treatment decisions and how these, in turn, influence outcomes and prognosis within the Lebanese cancer population. By also identifying barriers to NGS implementation and therapy access, the study seeks to inform future strategies to advance precision oncology in the region. Ultimately, our goal is to demonstrate the potential of NGS in optimizing personalized care and improving outcomes for sarcoma patients in the MENA context.

## 2. Materials and Methods

This retrospective real-world chart review study analyzed data from patients’ medical and electronic health records at the American University of Beirut Medical Center, a tertiary healthcare institution and major cancer referral center in Lebanon. The study included patients with a histologically confirmed diagnosis of bone or soft tissue sarcoma who underwent molecular profiling via NGS between January 2021 and December 2024. Patients with insufficient tumor tissue or failed sequencing were excluded. In the whole cohort, most patients underwent tissue-based sequencing, while only three were profiled using liquid biopsy.

Molecular profiling was performed using a combination of commercial and locally available platforms. The commercial platforms included assays from Foundation Medicine (Boston, MA, USA), namely *FoundationOne Liquid CDx*, which interrogates 324 genes, and *FoundationOne Heme*, which interrogates 406 genes and is specifically designed to include genes known to be somatically altered in hematologic malignancies and sarcomas. In addition, *Guardant360 TissueNext* (Guardant Health, Palo Alto, CA, USA) was used, which sequences 84 cancer-associated genes to identify somatic alterations. These commercial assays are considered CGP platforms and routinely report multiple classes of alterations (single-nucleotide variants, insertions/deletions, copy-number changes, and rearrangements), as well as TMB and MSI status.

Locally, sequencing was performed on the Illumina NextSeq 550 platform using the *AVENIO Roche assay* (Roche, Basel, Switzerland) which is compatible with FoundationOne and interrogates 324 cancer-related genes. This assay evaluates multiple variant classes, including single-nucleotide variants (SNVs), indels, fusions, and copy-number variations (CNVs), and also provides validated assessment of TMB and MSI. In addition, a sarcoma-specific targeted panel, *ARCHER FusionPlex* (63 genes; ArcherDx, Boulder, CO, USA) was used for detection of clinically relevant fusions and mutations; this assay does not provide TMB or MSI results.

This study was conducted in accordance with the ethical principles outlined in the Declaration of Helsinki (2013). Ethical approval was obtained from the Institutional Review Board at AUBMC (IRB ID: BIO-2025-0004), with a waiver of informed consent due to the retrospective nature of the study. All patient data were de-identified to maintain confidentiality, and no personally identifiable information was accessible to the researchers. Data collected included demographic characteristics, sarcoma subtype and staging, type of NGS test received, prior lines of systemic therapy, and disease status before and after NGS testing.

Early NGS was defined as molecular testing performed at diagnosis or prior to initiation of first-line systemic therapy. Late NGS was defined as testing performed after at least one prior line of systemic therapy or during disease progression in the metastatic setting. These definitions were applied consistently across all patients.

Genomic alterations were reported by the performing laboratories, which classify variants for actionability based on regulatory approvals, international clinical guidelines (e.g., NCCN, ESMO), and supporting peer-reviewed literature. In this study, actionability was further defined using a standardized framework integrating: (1) on-label regulatory approvals (FDA, EMA), (2) guideline-supported biomarker–therapy associations, and (3) strong biological or clinical evidence from peer-reviewed data.

Descriptive statistics were used to summarize patient characteristics. Continuous variables were reported as means ± standard deviations or medians with interquartile ranges (IQR), while categorical variables were presented as frequencies and percentages. Survival outcomes, including overall survival (OS) and progression-free survival (PFS), were estimated using the Kaplan–Meier method. Comparisons between patients who received NGS-based treatment adjustments (NBTA) and those who did not were made using the log-rank test. PFS was defined as the time from the date of NGS testing to the date of documented disease progression.

All data were managed using REDCap (Research Electronic Data Capture) version 15.9.1, and statistical analyses were performed using SPSS version 29. A *p*-value < 0.05 was considered statistically significant.

## 3. Results

### 3.1. Patient Characteristics

A total of 78 patients with histologically confirmed sarcoma underwent next-generation sequencing between January 2021 and December 2024. Baseline demographic and clinical characteristics according to NGS-guided treatment status are summarized in Table 1. The median age at diagnosis was 44 years (range: 8–81), with a predominance of males (60.3%, n = 47), while females accounted for 39.7% (n = 31). Most tumors were soft tissue sarcomas (70.5%, n = 55), with the remaining 29.5% (n = 23) classified as bone sarcomas.

The distribution of histologic subtypes is presented in Table 2. The most common subtypes were liposarcoma (17.9%), Ewing sarcoma (14.1%), leiomyosarcoma (11.5%), and osteosarcoma (11.5%). Less frequent subtypes included angiosarcoma, synovial sarcoma, pleomorphic undifferentiated sarcoma, chondrosarcoma, rhabdomyosarcoma, desmoplastic small round cell tumor (DSRCT), and unclassified sarcomas. The cohort was predominantly high-grade, with 75 patients harboring high-grade tumors, one intermediate-grade tumor, and two low-grade tumors. At initial diagnosis, 4 patients (5.1%) had stage I disease, 19 (24.4%) had stage II disease, 26 (33.3%) had stage III disease, and the remaining 29 patients (37.2%) presented with stage IV disease.

Systemic therapy prior to molecular profiling was documented in 64.1% of patients (n = 50), with a median of one prior line of treatment (range: 0–4). NGS was performed late in the disease course in the majority of patients (73.1%), whereas only 26.9% underwent mo-lecular testing early in their clinical trajectory.

### 3.2. Molecular Testing and Alterations

A total of 78 patients underwent molecular profiling using both commercial and locally available assays. FoundationOne Heme was performed in 61 patients (78.2%), of whom 21 (34.4%) had a targetable alteration identified. FoundationOne Liquid CDx was used in 3 patients (3.8%). Sequencing on the Illumina NextSeq 550 platform (AVENIO assay) was performed in 5 patients (6.4%), with one (20%) harboring a targetable alteration. Guardant360 TissueNext was used in 1 patient (1.3%) and identified a targetable alteration. ARCHER FusionPlex was performed in 8 patients (10.3%) but did not yield targetable findings.

The median number of mutations detected per patient was 3, ranging from 0 to 19. Subtype-level differences in mutation burden are shown in Table 2. Rhabdomyosarcoma and liposarcoma showed the highest median number of alterations per patient, while Ewing sarcoma demonstrated the lowest. MSI and TMB assessments were available for 67 patients. All were microsatellite stable (MSS), with no cases of microsatellite instability detected. Among the 67 with TMB results, 79% were classified as low, 19% as intermediate, and 2% as high.

**Figure 1:**
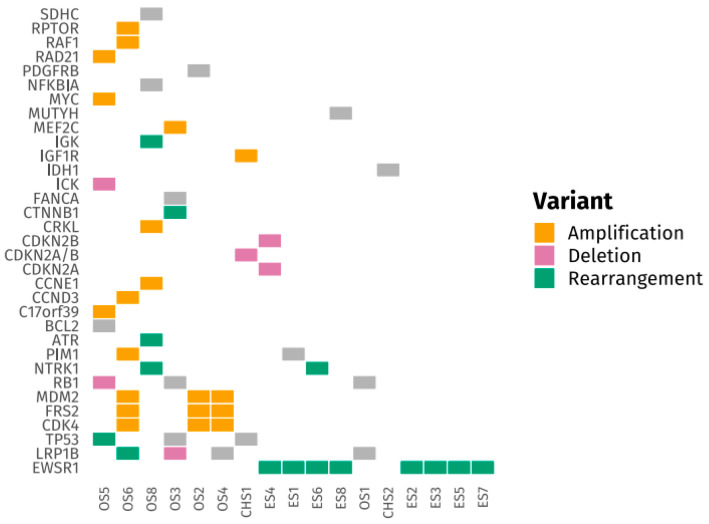
Tile Plot of Frequently Altered Genes in Bone Sarcomas (BS).

**Figure 2:**
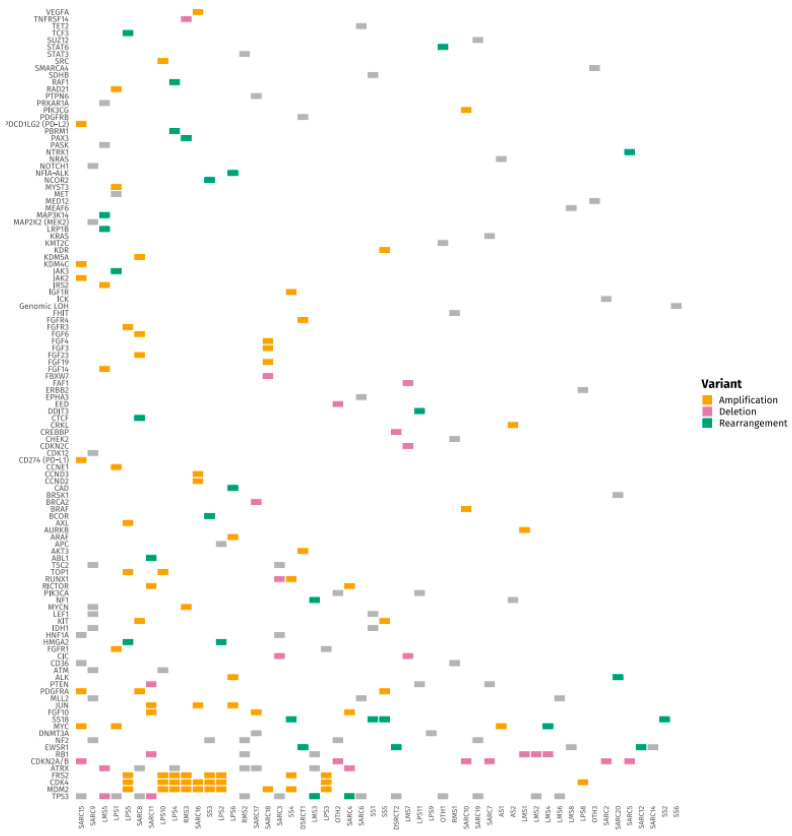
Tile Plot of Frequently Altered Genes in Soft Tissue Sarcomas (STS).

Figure 1 and Figure 2 present tile plots illustrating the most frequently altered genes in STS and BS, respectively. Alterations in *CDK4*, *PIK3CA*, *NF1*, and *ALK* genes were more commonly observed in STS, whereas BS samples exhibited fewer targetable alterations overall. Genomic alterations were frequently subtype-specific. *EWSR1* gene rearrangements were consistently observed in Ewing sarcoma and desmoplastic small round cell tumors. *CDK4* and *MDM2* gene amplifications were identified in liposarcoma and osteosarcoma. *TP53* and *RB1* gene mutations were common in leiomyosarcoma. Deletions in *CDKN2A/B* genes were noted in undifferentiated pleomorphic sarcoma and chondrosarcoma. *SS18* gene rearrangements were exclusively found in synovial sarcoma. Additional tile plots depicting the mutational landscape in individual subtypes, including Ewing sarcoma, osteosarcoma, liposarcoma, and leiomyosarcoma, are provided in Supplementary Figures S1–S4.

At the time of molecular profiling, targetable alterations were identified in 33% of patients. When reassessed with updated genomic knowledge and therapeutic approvals, this proportion increased to 42%.

### 3.3. Treatment Modifications and Drug Utilization

A total of 16 patients (20.5%) received a treatment adjustment based on NGS findings, referred to as NGS-Based Treatment Adjustment (NBTA). Among the remaining 62 patients who did not receive NBTA, the reasons included the absence of actionable targets in 67% of cases, the presence of no detectable mutations in 17%, and ineligibility due to clinical deterioration, financial constraints, or lack of drug access in 16%.

Among patients who received NBTA, responses varied. Partial responses were achieved in two cases: a liposarcoma patient with a BRCA2 mutation treated with PARP inhibitors (Olaparib and Talazoparib; PFS 29.5 months) and a patient with an NTRK1 fusion who received Larotrectinib (PFS 57.7 months). Stable disease was observed in several patients treated with ALK, CDK4/6, or PI3K/mTOR inhibitors, with PFS ranging from 5.2 to 20.5 months. However, the majority of patients (n = 8) experienced progressive disease as their best response, typically within the first 2–3 months of therapy. Access to targeted treatments was predominantly through self-funding, with only two patients able to secure drugs via compassionate access programs. These outcomes are summarized in Table 3.

### 3.4. Patients with Actionable Findings Who Did Not Receive Therapy

Despite the identification of actionable alterations or immunotherapy biomarkers, 10 patients did not receive NBTA. The reasons included rapid clinical deterioration, lack of access to targeted drugs, or ongoing response to standard therapy. These cases are detailed in Table 4.

### 3.5. Survival Outcomes

At the time of last follow-up, 68 patients (87.1%) had metastatic disease, and 30 patients (38.5%) had died. Among patients who received NBTA, the median PFS was 9.0 months compared with 2.0 months in patients who did not receive molecularly guided therapy (*p* = 0.023). Favorable outcomes within the NBTA group were primarily observed in a limited subset of patients with biologically well-defined, actionable alterations and access to matched therapies. These included a patient with liposarcoma harboring a BRCA2 mutation treated with PARP inhibitors (PFS 29.5 months) and a patient with an NTRK1 fusion treated with larotrectinib (PFS 57.7 months). Additional patients achieved disease stabilization with CDK4/6, ALK, or PI3K/mTOR inhibitors, with variable durations of benefit. In contrast, the majority of patients receiving NBTA experienced disease progression. Overall survival was numerically longer in the NBTA group (median 74.0 months vs. 48.0 months), although this difference did not reach statistical significance (*p* = 0.207).

## 4. Discussion

NGS has emerged as a transformative tool in the genomic characterization of sarcomas [2]. In our cohort, NGS provided valuable molecular insights across a broad range of histologic subtypes, including both common entities such as liposarcoma and Ewing sarcoma, as well as rare forms like undifferentiated pleomorphic sarcoma and synovial sarcoma. Consistent with global data, our findings reinforced the heterogeneity in the mutational landscape of sarcomas [6,11]. Subtype-specific alterations were observed, including *EWSR1* fusions in Ewing sarcoma and *CDK4* and *MDM2* amplifications in liposarcoma, reflecting well-established pathogenic mechanisms [12,13]. Additionally, the identification of potentially actionable mutations across multiple subtypes demonstrates the broad utility of NGS beyond diagnostic refinement. These findings echo international efforts highlighting the value of comprehensive genomic profiling in guiding precision oncology efforts for sarcoma patients [14].

Beyond validating known biological patterns, our results align closely with emerging international recommendations advocating for broad molecular characterization of mesenchymal tumors. The Italian Sarcoma Group’s 2025 consensus paper emphasizes that extended molecular profiling is now considered essential not only for therapeutic stratification but also for improving diagnostic accuracy in morphologically ambiguous sarcomas, particularly through the detection of structural variants, copy-number alterations, and gene fusions [15]. These capabilities were reflected in our cohort, where alterations such as CDK4/MDM2 amplifications, EWSR1 rearrangements, TP53/RB1 mutations, and CDKN2A/B deletions were consistently detected and, in several cases, directly informed treatment decisions. Importantly, the consensus recommends early molecular testing, ideally at diagnosis or first relapse, to maximize clinical utility. In contrast, 73% of patients in our cohort underwent NGS late in the disease course, highlighting a significant implementation gap between guideline recommendations and real-world practice in resource-limited settings.

Similarly, our findings parallel those of the ACC Sarcoma Working Group, which demonstrated the central role of NGS in identifying pathognomonic fusion transcripts across diverse sarcoma subtypes [16]. Systematic detection of canonical fusions such as EWSR1–FLI1, SS18–SSX, CIC–DUX4, and BCOR rearrangements has been shown to enhance diagnostic precision and may identify patients eligible for targeted or investigational therapies [17]. Fusion-driven tumors in our cohort, including Ewing sarcoma, desmoplastic small round cell tumor, and synovial sarcoma, were reliably detected, reinforcing fusion profiling as a cornerstone of modern sarcoma diagnostics.

The choice of NGS platform remains an important and unresolved question in sarcoma care. Sarcomas are characterized by complex genomic architecture, often driven by structural variants rather than recurrent point mutations. As such, platforms limited to hotspot mutation detection may be insufficient. Current evidence supports the use of hybrid-capture, DNA-based panels capable of detecting copy-number alterations and structural rearrangements, complemented by RNA sequencing when feasible, to optimize fusion detection [16]. In this context, both platforms used in our study (324- and 406-gene panels) were able to capture clinically relevant alterations; however, differences in gene content and fusion coverage may influence diagnostic yield.

Whole-genome sequencing (WGS), while comprehensive, is not currently necessary for routine clinical sarcoma management due to cost, data complexity, and limited incremental therapeutic yield over large, targeted panels in most cases [18]. Instead, disease-focused large panels (≥300 genes) represent a pragmatic balance between breadth and feasibility, particularly in centers with constrained resources.

Resource availability should guide platform selection. In high-income settings, integrated DNA/RNA profiling with broad panels or whole-exome sequencing may be justified, particularly for rare or diagnostically challenging sarcomas. In contrast, in low- and middle-income countries, including the MENA region, a stepwise approach, starting with targeted panels enriched for sarcoma-relevant genes and fusions, may offer the greatest clinical value while maintaining cost-effectiveness.

Panel design may also be tailored according to sarcoma subtype. While bone and soft-tissue sarcomas share overlapping genomic alterations, certain diagnoses warrant focused coverage (e.g., fusion-heavy panels for Ewing sarcoma, copy-number-oriented panels for liposarcoma). Nevertheless, given the frequent diagnostic uncertainty at presentation, a unified sarcoma panel covering both bone and soft-tissue entities is generally preferable in routine practice [19].

Importantly, our study highlights the tangible impact of NGS on clinical decision-making in real-world sarcoma care. In 20.5% of patients, NGS findings directly informed a change in management, resulting in next-generation sequencing-based treatment adjustment (NBTA), a rate comparable to or slightly higher than those reported in retrospective series from other academic centers [20,21,22,23]. Survival outcomes were analyzed descriptively. Patients who received NBTA demonstrated a longer median progression-free survival (9.0 months) compared with those who did not receive NGS-guided therapy (2.0 months). However, given the small NBTA cohort, histologic heterogeneity, and the inherent non-comparability of treatment groups, these findings should be interpreted cautiously and considered exploratory and hypothesis-generating rather than confirmatory. Overall survival differences did not reach statistical significance, although a numerical trend favoring NBTA was observed. Together, these observations suggest a potential clinical relevance of integrating molecular profiling into therapeutic planning and highlight the need for adequately powered prospective studies to define the true impact of NGS-guided interventions in sarcoma care [24,25].

Despite identifying actionable alterations in 33–42% of patients, only 20.5% ultimately received matched therapies. This gap reflects persistent real-world barriers, including limited access to targeted agents, high drug costs, rapid clinical deterioration following late testing, and infrastructure constraints affecting turnaround time and multidisciplinary interpretation [26,27]. Addressing these challenges will be critical to translating molecular insights into meaningful clinical benefit. Potential strategies include regional genomic databases, expansion of compassionate-use programs, and formal molecular tumor boards to optimize treatment matching [28,29,30,31,32]. The financial burden of NGS testing remains a global concern due to inconsistent reimbursement policies.

This study represents one of the first real-world evaluations of precision oncology implementation in sarcoma care within the MENA region, where comprehensive molecular profiling remains underutilized [33]. Our findings demonstrate the feasibility of integrating NGS into routine clinical practice and highlight its potential clinical value even in resource-constrained settings. By providing real-world data from an underserved region, this study adds to the growing body of evidence supporting broader access to genomic testing and molecular diagnostics as a critical component of equitable cancer care worldwide [34].

Nevertheless, several limitations must be acknowledged. The retrospective design and modest sample size may constrain the generalizability of our findings. Additionally, molecular profiling was frequently performed late in the disease course, reducing the likelihood of patients benefiting from genomic-guided therapies. Variability in the NGS assays used (FoundationOne vs. Guardant360) may also introduce inconsistencies in mutation detection and reporting. Furthermore, access to targeted agents was often restricted by cost and limited availability, which likely led to an underestimation of the true clinical impact of NGS-guided treatment in this cohort.

Looking forward, our findings support future initiatives aimed at integrating molecular testing earlier in the treatment trajectory, ideally at diagnosis or after the first line of therapy [33]. Prospective studies are needed to clarify the survival impact of NBTA and to define optimal sequencing strategies. International collaboration will be essential to harmonize panel design, improve access to targeted agents, and ensure equitable implementation of precision oncology across diverse healthcare systems [35].

## Figures and Tables

**Table 1:** Baseline characteristics according to NGS-guided treatment (NBTA) status (N = 78).

Characteristic	No NBTA (n = 62)	NBTA (n = 16)	*p* Value
**Age at diagnosis, mean ± SD (years)**	41.68 ± 20.69	44.38 ± 14.73	**0.555**
**Sex**			**0.713**
Female	24 (38.7%)	7 (43.8%)	
Male	38 (61.3%)	9 (56.3%)	
**Sarcoma type**			**0.128**
Bone	21 (33.9%)	2 (12.5%)	
Soft tissue	41 (66.1%)	14 (87.5%)	
**Stage at diagnosis**			**0.258**
Localized (Stages I–III)	37 (59.7%)	12 (75%)	
Metastatic (Stage IV)	25 (40.3%)	4 (25%)	
**Tumor grade**			**0.503**
High grade	60 (96.8%)	15 (93.8%)	
Low/Intermediate grade	2 (3.2%)	1 (6.3%)	
**Prior systemic treatment before profiling**			**0.881**
No	22 (35.5%)	6 (37.5%)	
Yes	40 (64.5%)	10 (62.5%)	
**Prior systemic therapy lines**			**0.087**
0–1 line	36 (58.1%)	13 (81.3%)	
≥2 lines	26 (41.9%)	3 (18.8%)	
**Timing of molecular profiling**			**0.210**
Early	19 (30.6%)	2 (12.5%)	
Late	43 (69.4%)	14 (87.5%)	

**Table 2:** Median Number of Genomic Alterations per Patient by Sarcoma Histological Subtype.

Histological Subtype	Number of Patients	Median Alterations Detected per Patient (Range)
Angiosarcoma	5	2 (0–19)
Chondrosarcoma	3	3 (1–4)
Desmoplastic Small Round Cell Tumor	2	3.5 (3–4)
Ewing Sarcoma	11	1 (1–4)
Leiomyosarcoma	9	2 (2–8)
Liposarcoma	14	4.5 (0–8)
Osteosarcoma	9	4 (0–8)
Pleomorphic Undifferentiated Sarcoma (PUS)	3	3 (0–10)
Rhabdomyosarcoma	3	5 (3–6)
Sarcoma (Unspecified)	13	3 (0–8)
Synovial Sarcoma	6	4 (0–5)

**Table 3:** Actionable Genomic Alterations and Clinical Outcomes of Targeted Therapies Across Sarcoma Subtypes (n = 78).

Histological Subtype	Actionable Mutations	Treatment	Best Response	Access	PFS (Months)
Chondrosarcoma	PIK3CA, NF2	Everolimus	PD	Self-Funding	1.4
Leiomyosarcoma	NF1	Trametinib	PD	Self-Funding	5.5
Liposarcoma	ALK	Crizotinib	SD	Self-Funding	5.2
Liposarcoma	BRCA2	Olaparib Talazoparib	PR	Compassionate access Self-Funding	29.5
Liposarcoma	CDK4	Palbociclib	PD	Self-Funding	1.9
Liposarcoma	CDK4	Ribociclib	SD	Self-Funding	7.0
Liposarcoma	CDK4	Abemaciclib	SD	Self-Funding	15.6
Liposarcoma	CDK4	Abemaciclib	SD	Self-Funding	20.5
Liposarcoma	MET	Cabozantinib Crizotinib	PD	Self-Funding	3.3
Liposarcoma	PIK3CA, PTEN	Everolimus	PD	Self-Funding	1.8
Liposarcoma	CDK4	Abemaciclib	PD	Self-Funding	0.9
Osteosarcoma	CDK4	Palbociclib	PD	Self-Funding	0.7
Sarcoma	ALK	Alectinib	SD	Self-Funding	6.8
Sarcoma	NTRK1	Lortrectinib	PR	Compassionate access	57.7
Sarcoma	PTEN	Everolimus	SD	Self-Funding	7.3
Synovial Sarcoma	KIT, PDGFRA	Imatinib	PD	Self-Funding	2.2

Abbreviations: PD, progressive disease; PFS, progression-free survival; PR, partial response; SD, stable disease.

**Table 4:** Patients with Actionable Mutations or Immunotherapy Biomarkers Who Did Not Receive Molecularly Directed Treatment.

Histological Subtype	Actionable Mutations	Reasons Not Pursued
Angiosarcoma	TMB High	Rapid Progression
Angiosarcoma	NRAS	Rapid Progression
Desmoplastic Small Round Cell Tumor	PDGFRB	No Access to Medication
Ewing Sarcoma	NTRK1	No Access to Medication
Liposarcoma	ATM, CDK4	No Access to Medication
Liposarcoma	CDK4, NF2	No Access to Medication
Osteosarcoma	NTRK1	Responding to Standard Therapy
Pleomorphic Undifferentiated Sarcoma (PUS)	CD274 (PD-L1), PDCD1LG2 (PD-L2), PDGFRA	Rapid Progression
Sarcoma	KIT, PDGFRA	Rapid Progression
Sarcoma	TSC2	Responding to Standard Therapy

Abbreviations: TMB, tumor mutational burden.

## Data Availability

The original contributions presented in this study are included in the article/Supplementary Materials. Further inquiries can be directed to the corresponding author.

## Supplementary Materials

The following supporting information can be downloaded at https://www.mdpi.com/article/10.3390/medsci14010046/s1, Figure S1: Tile Plot of Frequently Altered Genes in Ewing Sarcoma; Figure S2: Tile Plot of Frequently Altered Genes in Osteosarcoma; Figure S3: Tile Plot of Frequently Altered Genes in Liposarcoma; Figure S4: Tile Plot of Frequently Altered Genes in Leiomyosarcoma.
