# Supplementary material for: Real-World Implementation of Next-Generation Sequencing in Sarcoma: Molecular Insights and Therapeutic Outcomes
Source: Med Sci (Basel). 2026 Jan 17;14(1):46. doi: 10.3390/medsci14010046 (PMC12821713; doi:10.3390/medsci14010046)

Supplementary Figure S1. Tile Plot of Frequently Altered Genes in Ewing Sarcoma.

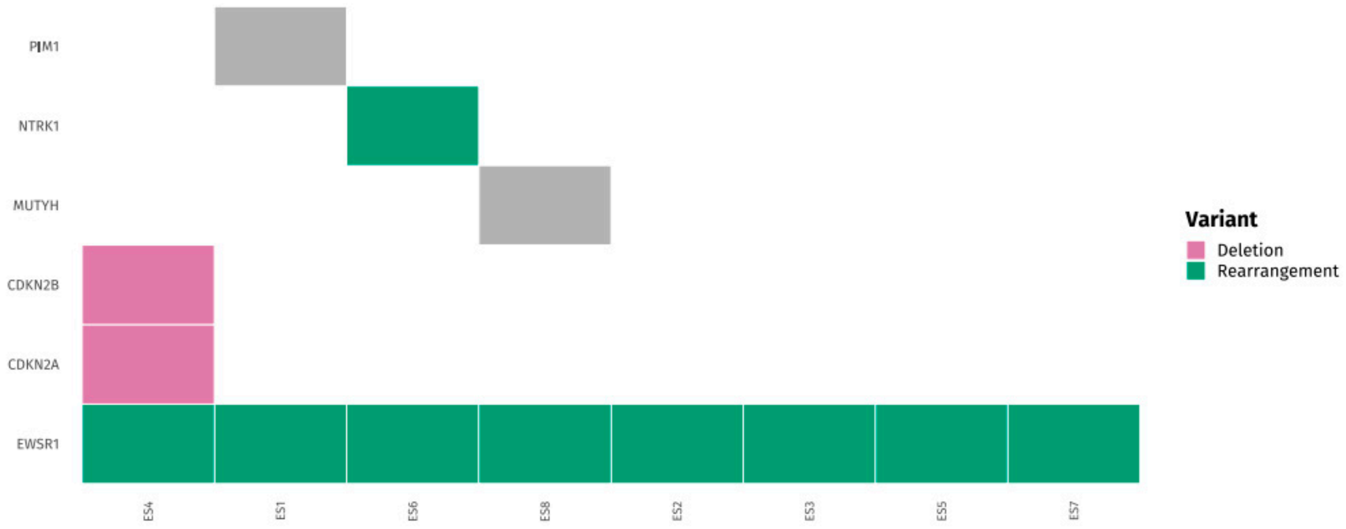

Supplementary Figure S2. Tile Plot of Frequently Altered Genes in Osteosarcoma.

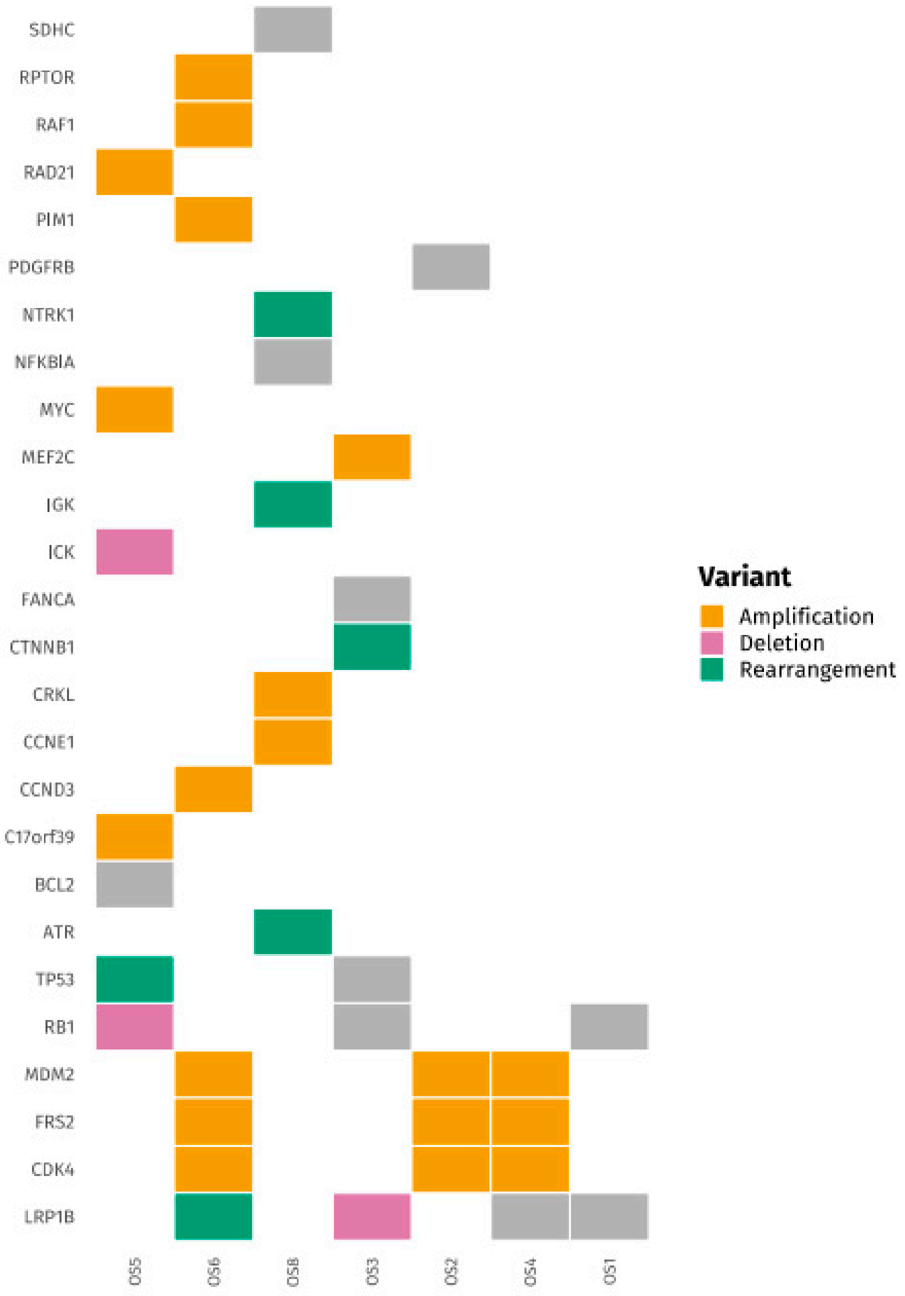

Supplementary Figure S3. Tile Plot of Frequently Altered Genes in Liposarcoma.

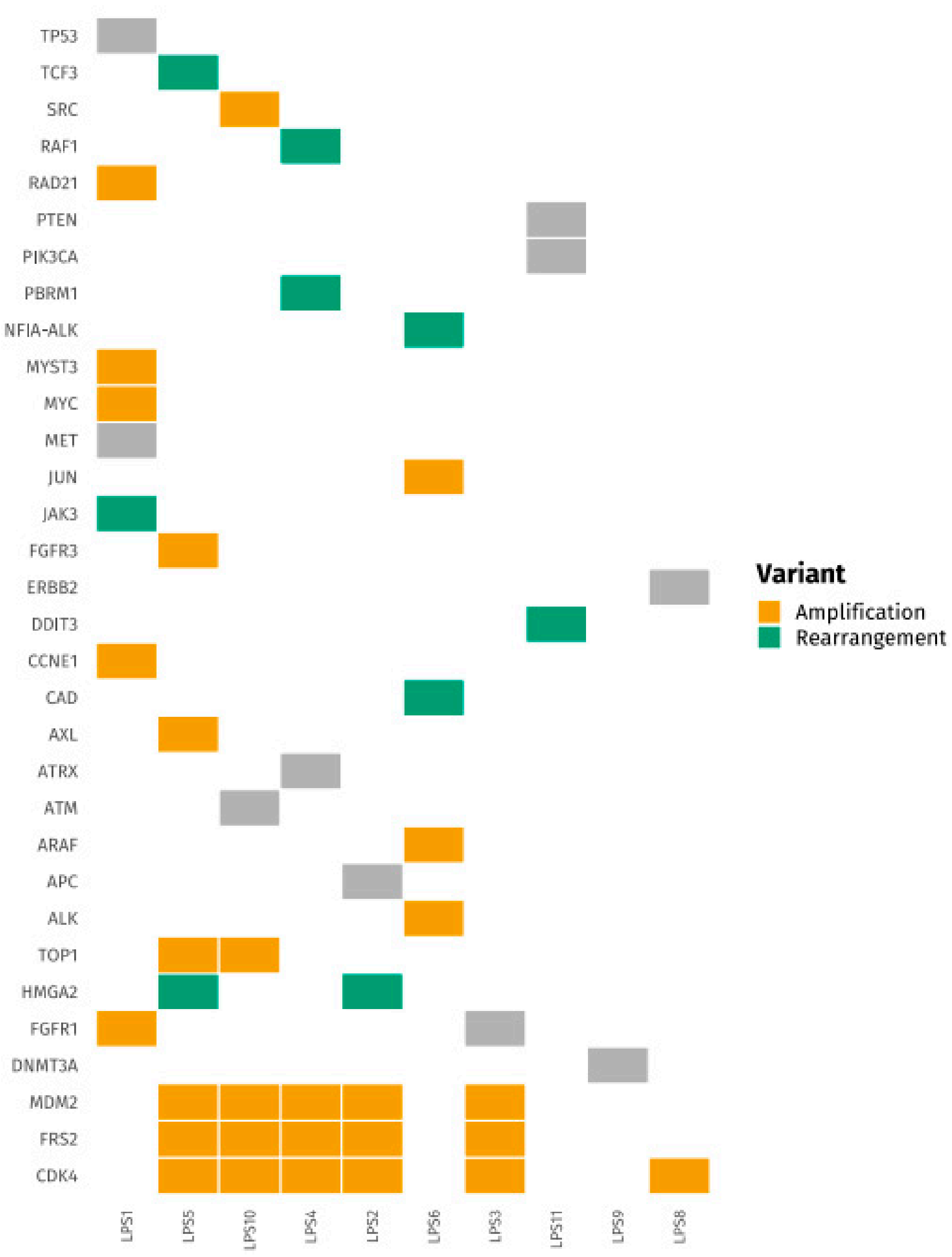

Supplementary Figure S4. Tile Plot of Frequently Altered Genes in Leiomyosarcoma.

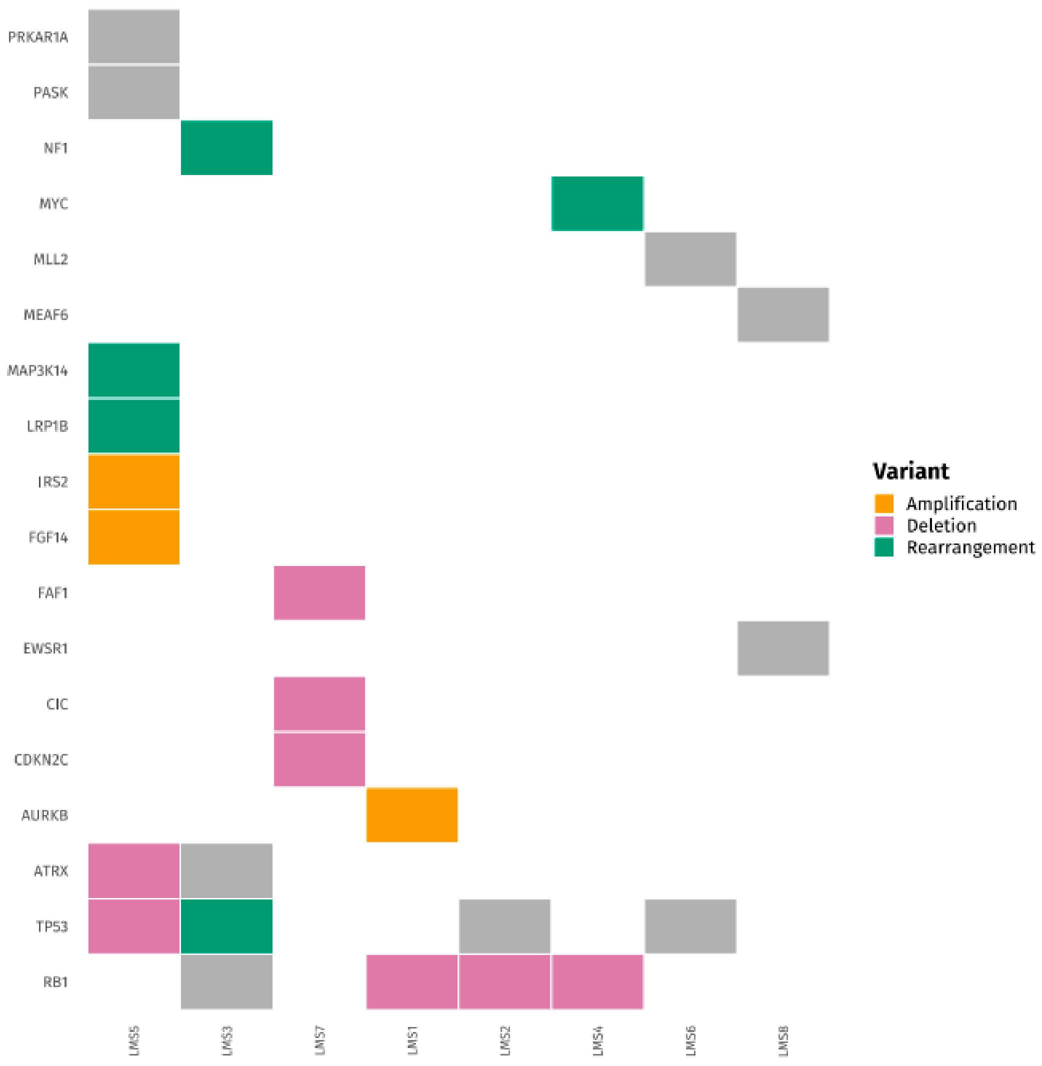

Supplement: Supplementary file 1 [file medsci-14-00046-s001.zip › medsci-4023545-supplementary.pdf]
